# Use of No-Cost Preventive Services Jeopardized by *Kennedy v Braidwood*

**DOI:** 10.1001/jamahealthforum.2025.1559

**Published:** 2025-04-17

**Authors:** Michelle Bronsard, Adrienne Sabety, Minttu Rönn, Nicole A. Swartwood, Joshua A. Salomon

**Affiliations:** 1 Department of Health Policy, Stanford University, Stanford, California; 2 Department of Global Health and Population, Harvard T.H. Chan School of Public Health, Boston, Massachusetts

## Abstract

This cross-sectional study analyzes the use of 10 no-cost preventive services by individuals with employer-sponsored health insurance, which may be threatened by the upcoming *Kennedy v Braidwood* Supreme Court case.

## Introduction

The Affordable Care Act (ACA) mandates that private insurers cover specific preventive services without cost sharing. In 2022, a Texas district court ruled in *Braidwood v Becerra* that mandating coverage of services recommended by the US Preventive Services Task Force (USPSTF) was unconstitutional and barred mandate enforcement for post-ACA USPSTF recommendations.^1^ The ruling was partially upheld on 2024 appeal, and the Supreme Court will hear the case (now *Kennedy v Braidwood*) in April 2025.

Prior studies have estimated how many people receive any ACA-mandated services^2^ or selected USPSTF-recommended services.^3^ Here, we assess how rolling back the ACA mandate in *Braidwood* or subsequent cases could directly impact privately insured individuals by estimating how many enrollees have received, at no cost, any of 10 preventive services jeopardized by this case.

## Methods

This cross-sectional study using deidentified data received approval from Stanford University’s institutional review board. Reporting followed STROBE guidelines. The sample comprised all individuals aged 15 to 64 years enrolled in employer-sponsored health insurance (ESHI) as of 2018, reported in the Merative MarketScan Commercial Database.^4^ We weighted the sample to represent the ESHI enrollee population in 2018 by state, sex, and age (eTables 1-2 in Supplement 1).

We selected 10 preventive services potentially impacted by *Braidwood* due to receiving new or revised USPSTF A or B recommendations since March 2010. Selected services include statin use to prevent cardiovascular disease; preexposure prophylaxis for HIV; medication to reduce breast cancer risk; and new or expanded screenings for breast, cervical, colorectal, and lung cancers and hepatitis B virus, hepatitis C virus, and HIV infections. We specified inclusion and exclusion criteria to identify USPSTF-recommended service use covered by the mandate (eTable 3 in Supplement 1). Between October 2023 and February 2025, we analyzed inpatient, outpatient, and prescription claims and payment data recorded from January 2018 to December 2022 (Redivis [RRID:SCR_023111]). We report counts and proportions of enrollees receiving at least 1 service without cost sharing nationally and by sex, age, and state.

## Results

The sample comprised 16.1 million enrollees in MarketScan, representing 130.9 million ESHI enrollees in 2018. Approximately 30% of enrollees (39.1 million individuals) nationally received at least 1 of the 10 preventive services without cost sharing from 2018 to 2022 (Table 1). Among enrolled women, 46.1% received at least 1 no-cost service. Nationally, the most widely received services were screenings for cervical cancer (23.6 million), hepatitis C virus infection (11.1 million), and HIV infection (10.5 million). Across states, proportions of enrollees receiving no-cost services ranged from 20.5% to 38.5% (Table 2). Thirteen states had at least 1 million no-cost service recipients, including more than 3 million in Texas, where *Braidwood* originated.

**Table 1.** Individuals With ESHI Receiving at Least 1 No-Cost Preventive Service Jeopardized by *Kennedy v Braidwood* by Age and Sex, 2018-2022^a^

Variable	ESHI enrollees, No. in thousands	ESHI enrollees receiving no-cost services, No. in thousands	Proportion of ESHI enrollees receiving no-cost services, %
Enrollees in 2018	130 952	39 097	29.9
By age group, y			
15-24	24 528	4101	16.7
25-34	26 772	8112	30.3
35-44	26 580	9656	36.3
45-54	27 295	10 901	39.9
55-64	25 777	9095	35.3
By sex			
Women	65 914	30 366	46.1
Men	65 038	8731	13.4

Abbreviation: ESHI, employer-sponsored health insurance.

^a^ Individuals with ESHI were defined as those enrolled in ESHI in 2018. These estimates were derived from MarketScan claims data, with counts weighted using estimates of the population with ESHI by state, sex, and age from the 2018 American Community Survey.

**Table 2.** Individuals With ESHI Receiving at Least 1 No-Cost Preventive Service Jeopardized by *Kennedy v Braidwood* by State, 2018-2022^a^

State	ESHI enrollees, No. in thousands	ESHI enrollees receiving no-cost services, No. in thousands	Proportion of ESHI enrollees receiving no-cost services, %
Alaska	277	57	20.5
Alabama	1899	568	29.9
Arkansas	1058	233	22.0
Arizona	2571	656	25.5
California	15 114	4815	31.9
Colorado	2389	694	29.1
Connecticut	1564	443	28.3
District of Columbia	319	115	35.9
Delaware	400	118	29.4
Florida	7075	2226	31.5
Georgia	4168	1295	31.1
Hawaii	611	178	29.2
Iowa	1398	306	21.9
Idaho	659	141	21.4
Illinois	5520	1571	28.5
Indiana	2886	818	28.4
Kansas	1248	354	28.3
Kentucky	1720	527	30.7
Louisiana	1616	539	33.4
Massachusetts	3141	1209	38.5
Maryland	2681	948	35.4
Maine	538	142	26.3
Michigan	4202	1458	34.7
Minnesota	2566	827	32.2
Missouri	2560	693	27.1
Mississippi	1062	247	23.3
Montana	378	95	25.1
North Carolina	4036	1267	31.4
North Dakota	334	81	24.3
Nebraska	832	191	22.9
New Hampshire	641	182	28.4
New Jersey	4016	1348	33.6
New Mexico	647	136	21.0
Nevada	1181	255	21.6
New York	7980	2404	30.1
Ohio	4909	1339	27.3
Oklahoma	1452	299	20.6
Oregon	1651	550	33.3
Pennsylvania	5541	1632	29.5
Rhode Island	464	141	30.4
South Carolina	1912	539	28.2
South Dakota	351	97	27.8
Tennessee	2615	717	27.4
Texas	10 693	3215	30.1
Utah	1400	304	21.7
Virginia	3652	1193	32.7
Vermont	260	66	25.2
Washington	3190	801	25.1
Wisconsin	2660	850	32.0
West Virginia	672	168	25.1
Wyoming	245	50	20.5
All states	130 952	39 097	29.9

Abbreviation: ESHI, employer-sponsored health insurance.

^a^ Individuals with ESHI were defined as those enrolled in ESHI in 2018. These estimates were derived from MarketScan claims data, with counts weighted using estimates of the population with ESHI by state, sex, and age from the 2018 American Community Survey.

## Discussion

This cross-sectional study presented a detailed, comprehensive assessment of ACA-mandated no-cost preventive service use potentially jeopardized by *Braidwood* and future challenges. Among ESHI enrollees in 2018 aged 15 to 64 years, nearly 1 in 3 (and nearly half of women) received no-cost preventive services from 2018 to 2022 covered under the ACA mandate but threatened by *Braidwood*. While results varied across states, proportions were higher than 20% in every state.

Our estimates may be considered lower bounds for several reasons. Some jeopardized services (eg, anxiety screenings) were not analyzed because of difficult discernment in claims. Individuals enrolled in 2018 could be right-censored in the data due to changes in either individual employment status or data provider participation in MarketScan, biasing cumulative estimates downward. We included only people aged 15 to 64 years, consistent with the focus on ESHI and USPSTF recommendations covering adolescents and adults, but potentially excluding some services received by children or older adults. Another limitation was absence of data relevant to evaluating health disparities.

Extensive literature indicates that providing preventive services without cost sharing can increase uptake.^5^ The ACA preventive services mandate has been consistently popular in public opinion polls.^6^ Removing the no-cost coverage requirement would affect millions of individuals who benefit from preventive services across all states. Potential downstream consequences should be considered, including delayed health care access and increased morbidity, mortality, and disparities.

## Notes

Supplement 1. eTable 1. Characteristics of the study sample of enrollees in employer-sponsored health insurance in 2018 eTable 2. Study sample of enrollees in employer-sponsored health insurance in 2018, by state eTable 3. Inclusion and exclusion criteria used to identify recipients of preventive services jeopardized by *Kennedy v Braidwood* eReferences

Supplement 2. Data Sharing Statement
