# Error in Discussion

**DOI:** 10.1001/jamahealthforum.2025.2210

**Published:** 2025-05-30

The Research Letter titled "Use of No-Cost Preventive Services Jeopardized by *Kennedy v Braidwood*,”^1^ published online on April 17, 2025, was corrected to fix an error in the Discussion. In the second sentence of the Discussion, “aged 18 to 64 years” should have been “aged 15 to 64 years.” This article has been corrected online.
