# Supplementary material for: Use of No-Cost Preventive Services Jeopardized by Kennedy v Braidwood
Source: JAMA Health Forum. 2025 Apr 17;6(4):e251559. doi: 10.1001/jamahealthforum.2025.1559 (PMC12006864; doi:10.1001/jamahealthforum.2025.1559)
Supplement: Supplement 1. — eTable 1. Characteristics of the study sample of enrollees in employer-sponsored health insurance in 2018 eTable 2. Study sample of enrollees in employer-sponsored health insurance in 2018, by state eTable 3. Inclusion and exclusion criteria used to identify recipients of preventive services jeopardized by Kennedy v Braidwood eReferences [file jamahealthforum-e251559-s001.pdf]

Supplemental Online Content

Bronsard M, Sabety A, Rönn M, Swartwood NA, Salomon JA. Use of no-cost preventive services jeopardized by *Kennedy v Braidwood*. *JAMA Health Forum*. 2025;6(4):e251559. doi:10.1001/jamahealthforum.2025.1559

eTable 1. Characteristics of the study sample of enrollees in employer-sponsored health insurance in 2018

eTable 2. Study sample of enrollees in employer-sponsored health insurance in 2018, by state

eTable 3. Inclusion and exclusion criteria used to identify recipients of preventive services jeopardized by *Kennedy v Braidwood*

eReferences

This supplemental material has been provided by the authors to give readers additional information about their work.

© 2025 Bronsard M et al. *JAMA Health Forum*.

eTable 1. Characteristics of the study sample of enrollees in employer-sponsored health insurance in 2018

	Unweighted count	%	Weighted count	%
Enrollees in 2018	16,083,239	100.0	130,952,051	100.0
By age group				
15-24	3,020,713	18.8	24,528,070	18.7
25-34	3,794,497	23.6	26,771,576	20.4
35-44	3,282,932	20.4	26,580,447	20.3
45-54	3,191,626	19.8	27,295,380	20.8
55-64	2,793,471	17.4	25,776,577	19.7
By sex				
Women	8,256,302	51.3	65,914,227	50.3
Men	7,826,937	48.7	65,037,824	49.7
By years of observation*				
1 or more	16,083,239	100.0	130,952,051	100.0
2 or more	9,861,589	61.3	82,137,722	62.7
3 or more	6,466,863	40.2	54,616,017	41.7
4 or more	4,650,498	28.9	39,727,445	30.3
5	2,678,414	16.6	23,023,645	17.6

Source: MarketScan claims data weighted using estimates of the population with employer-sponsored health insurance by state, sex, and age from the 2018 American Community Survey.[1]

* Study sample defined as those observed in the dataset during 2018. Right censoring in the data can result from changes in individual insurance status or changes in providers contributing data to MarketScan.

© 2025 Bronsard M et al. *JAMA Health Forum*.

eTable 2. Study sample of enrollees in employer-sponsored health insurance in 2018, by state

State	Unweighted count	Weighted count
Alaska	15,506	277,452
Alabama	209,945	1,899,083
Arkansas	62,963	1,058,070
Arizona	307,550	2,571,316
California	1,393,153	15,113,695
Colorado	292,280	2,388,626
Connecticut	370,730	1,564,378
District of Columbia	27,702	319,282
Delaware	79,383	399,828
Florida	1,389,488	7,075,206
Georgia	532,164	4,167,675
Hawaii	2,291	610,666
Iowa	139,213	1,398,296
Idaho	46,433	659,187
Illinois	604,451	5,519,716
Indiana	298,189	2,885,757
Kansas	170,028	1,248,250
Kentucky	264,970	1,719,959
Louisiana	188,190	1,616,474
Massachusetts	250,042	3,140,758
Maryland	279,129	2,680,633
Maine	40,340	538,166
Michigan	516,608	4,201,907
Minnesota	212,207	2,565,778
Missouri	345,231	2,559,669
Mississippi	133,555	1,061,703

© 2025 Bronsard M et al. *JAMA Health Forum*.

State	Unweighted count	Weighted count
Montana	19,595	378,016
North Carolina	533,432	4,035,543
North Dakota	11,394	333,779
Nebraska	88,371	832,057
New Hampshire	40,248	641,219
New Jersey	567,408	4,016,165
New Mexico	38,819	646,863
Nevada	123,926	1,180,965
New York	1,351,088	7,980,014
Ohio	724,954	4,908,840
Oklahoma	233,655	1,451,584
Oregon	145,160	1,651,495
Pennsylvania	605,137	5,540,953
Rhode Island	35,817	463,831
South Carolina	271,111	1,912,062
South Dakota	23,317	350,732
Tennessee	272,947	2,614,562
Texas	1,563,073	10,692,731
Utah	120,133	1,399,809
Virginia	444,270	3,652,164
Vermont	10,306	259,983
Washington	306,420	3,190,066
Wisconsin	318,589	2,659,884
West Virginia	47,796	672,135
Wyoming	14,532	245,070

Source: MarketScan claims data weighted using estimates of the population with employer-sponsored health insurance by state, sex, and age from the 2018 American Community Survey.[1]

© 2025 Bronsard M et al. *JAMA Health Forum*.

eTable 3. Inclusion and exclusion criteria used to identify recipients of preventive services jeopardized by *Kennedy v Braidwood*[2-5]

USPSTF service recommended with A or B grade	Inclusion codes for service recipients (code type and code number)*	Exclusion codes for service recipients (code type and code number)*†	Change in USPSTF recommendation since 2010 (year of change)	If Braidwood upheld, is service still protected under no-cost mandate?‡	Criteria for counting service recipients in this analysis
Breast cancer: medication use to reduce risk	NDC: Multiple (106 codes)§	ICD-10: Z85.3<br>ICD-9: V10.3	Recommendation to offer (updated from prior recommendation to "discuss") risk-reducing medications for women at increased risk for breast cancer (2013)	No longer protected	Women 35-64 yrs excluding those with code(s) indicating a history of breast cancer
Breast cancer: screening	CPT: 76083, 76085, 76092, 77052, 77057, 77063, 77067<br>HCPCS: G0202, G0203	ICD-10: D24.X, N60.8X, N60.9X, Z15.01, Z85.3<br>ICD-9: 174.X, 217.X, V10.3, V84.01	Updated to extend to ages 40-49 yrs (2024)	No longer protected for ages 40-49 yrs	Women 40-49 yrs excluding those with code(s) indicating history of breast cancer or genetic risk factors

© 2025 Bronsard M et al. *JAMA Health Forum*.

USPSTF service recommended with A or B grade	Inclusion codes for service recipients (code type and code number)*	Exclusion codes for service recipients (code type and code number)*†	Change in USPSTF recommendation since 2010 (year of change)	If Braidwood upheld, is service still protected under no-cost mandate?‡	Criteria for counting service recipients in this analysis
Cervical cancer: screening	CPT: 87620-87622, 87624, 87625, 88141-88143, 88147, 88148, 88150, 88152, 88153, 88164-88167, 88174, 88175, 0500T<br>HCPCS: G0101, G0123, G0124, G0141, G0143, G0144, G0145, G0147, G0148, P3000, P3001, Q0091, G0476<br>ICD-10: Z11.51<br>ICD-9: V73.81	CPT: 57530, 57531, 57540, 57545, 57550, 57555, 57556, 58150, 58152, 58200, 58210, 58240, 58260, 58262, 58263, 58267, 58270, 58275, 58280, 58285, 58290-58294, 58548, 58550, 58552-58554, 58570-58573, 58575, 58951, 58953, 58954, 59856, 59135<br>ICD-10: 0UTC0ZZ, 0UTC4ZZ, 0UTC7ZZ, 0UTC8ZZ, Q51.5, Z90.710, Z90.712, C53.X<br>ICD-9: 75243, 674, 180.X, V88.03, V88.01	Updated to include all women 21-65 (from prior recommendation to screen sexually active women), and specify screening frequency and technologies (2012)	HPV testing and newer screening technologies no longer protected	Women 21-64 yrs excluding those with code(s) indicating history of cervical cancer or no cervix
Colorectal cancer: screening	CPT: 45330, 45331, 45338, 45346, 45378, 45380, 45381, 45384, 45385, 45388, 74261-74263, 81528, 82270, 82274<br>HCPCS: G0104, G0105, G0107, G0121, G0328	CPT: 44150-44158, 44210-44212<br>ICD-10: 0DTE8ZZ, 0DTE7ZZ, 0DTE4ZZ, 0DTE0ZZ, C18.X, C19.X, C20.X, C21.2, C21.8, C78.5, Z85.038, Z85.048, Z86.010<br>ICD-9: 4581-4583, 153.X, 154.0, 154.1, 197.5, V10.05, V10.06, V12.72	New recommendation for ages 45-49 years (2021)	No longer protected for ages 45-49 yrs	Individuals 45-49 yrs excluding those with code(s) indicating history of colorectal cancer or polyps

© 2025 Bronsard M et al. *JAMA Health Forum*.

USPSTF service recommended with A or B grade	Inclusion codes for service recipients (code type and code number)*	Exclusion codes for service recipients (code type and code number)*†	Change in USPSTF recommendation since 2010 (year of change)	If Braidwood upheld, is service still protected under no-cost mandate?‡	Criteria for counting service recipients in this analysis
Hepatitis B virus infection in adolescents and adults: screening	CPT: 86704, 86706, 87340, 87341, 87515, 87517<br>HCPCS: G0499	ICD-10: B16.X, B17.0, B18.0, B18.1, B19.1X<br>ICD-9: 070.2X, 070.3X	Upgraded from D to B recommendation for screening among persons at "high risk" for infection (2014) or subsequently adolescents or adults at "increased risk" for infection (2020)	No longer protected	Individuals 15-64 yrs excluding those with code(s) indicating history of acute or chronic HBV
Hepatitis C virus infection in adolescents and adults: screening	CPT: 80074, 86803, 86804, 87520-87522, 87902<br>HCPCS: G0472, 3266F	ICD-10: B17.1X, B18.2, B19.2X, K70.1X<br>ICD-9: V02.62, 070.4X, 070.5X, 070.7X	Upgraded from D to B recommendation for screening adults born between 1945 and 1965 and others at high risk (2013), and subsequently updated to recommend screening for all adults aged 18 to 79 years (2020)	No longer protected	Individuals 18-64 yrs excluding those with code(s) indicating history of acute or chronic HCV
Human immunodeficiency virus (HIV) infection: screening	CPT: 86689, 86701-86703, 87389-87391, 87534-87539, 87806<br>HCPCS: G0432, G0433, G0435, G0437, G0298, G0475, S3645<br>ICD-10: Z11.4	ICD-10: B20.X, B21.X, B22.X, B23.X, B24, B97.35, Z21<br>ICD-9: 042, 043, 044, 07953, V08	Updated to extend to adolescents and adults who are not at "increased risk" for acquisition of HIV infection (2013)	No longer protected for adolescents and adults without "increased risk"	Individuals 15-64 yrs excluding those with code(s) indicating a history of HIV

© 2025 Bronsard M et al. *JAMA Health Forum*.

USPSTF service recommended with A or B grade	Inclusion codes for service recipients (code type and code number)*	Exclusion codes for service recipients (code type and code number)*†	Change in USPSTF recommendation since 2010 (year of change)	If Braidwood upheld, is service still protected under no-cost mandate?‡	Criteria for counting service recipients in this analysis
Lung cancer: screening	CPT: 71271<br>HCPCS: G0927, S8032	ICD-10: Z86.005, C34.9X, Z85.1X<br>ICD-9: 162.X, V10.11	New recommendation for screening at ages 55-80 (2013), subsequently expanded to 50-80 (2021)	No longer protected	Individuals 50-64 yrs excluding those with code(s) indicating a history of lung cancer
Prevention of acquisition of HIV: preexposure prophylaxis	NDC: Multiple (48 codes)§<br>ICD-10: Z29.81	ICD-10: B18.0, B18.1, B19.1X, B20.X, B21.X, B22.X, B23.X, B24, B97.35, Z21<br>ICD-9: 042, 043, 044, 07953, V08, 070.22, 070.23, 070.32, 070.33	New recommendation (2019)	No longer protected	Individuals 15-64 yrs excluding those with code(s) indicating history of HIV or chronic HBV‖
Statin use for the primary prevention of cardiovascular disease in adults: preventive medication	NDC: Multiple (2118 codes)§	ICD-10: I10.X, I11.X, I12.X, I13.X, I14.X, I15.X, I20.X, I21.X, I22.X, I23.X, I24.X, I25.X, I50.X, I60.X, I61.X, I62.X, 163.X, 165.X, 166.X<br>ICD-9: 401.X-405.X, 410.X-414.X, 428.X, 430.X-434.X	New recommendation (2016)	No longer protected	Individuals 40-64 yrs excluding those with code(s) indicating history of cardiovascular disease or familial hypercholesterolemia

* Codes for services and exclusionary diagnoses were identified using Current Procedural Terminology (CPT), Healthcare Common Procedure Coding System (HCPCS), National Drug Codes (NDC), and the ninth and tenth revisions of the *International Classification of Diseases* (ICD).[6-11]
† For each service, an enrollee was considered a non-recipient of the service if any exclusionary code was observed prior to the time of service receipt between 2011 and 2022.
‡ "Protected" refers to services that are mandated for no-cost coverage under the Affordable Care Act.
§ Complete list of codes available at github.com/PPML/preventive_services/
|| History of chronic HBV was an exclusion criterion to avoid false-positive identification of HIV PrEP recipients where tenofovir used to treat HBV infection.

© 2025 Bronsard M et al. *JAMA Health Forum*.

eReferences

1. Ruggles S, Flood S, Sobek M, Backman D, Chen A, Cooper G, Richards S, Rodgers R, Schouweiler M. IPUMS USA: Version 15.0 [Dataset]. IPUMS; 2024. https://doi.org/10.18128/D010.V15.0
2. University of Michigan V-BID Center. Clinical and equity implications of Braidwood v. Becerra. Published June 2, 2023. Accessed March 26, 2025. https://vbidcenter.org/clinical-and-equity-implications-of-braidwood-v-becerra/
3. United States of Care. Braidwood Management Inc. v. Becerra overturns Affordable Care Act's no-cost mandate for many preventive services. Published 2023. Accessed March 26, 2025. https://unitedstatesofcare.org/wp-content/uploads/2023/09/US-of-Care-FACT-SHEET-Braidwood-Management-Inc.-v.-Becerra-Overturns-Affordable-Care-Acts-No-Cost-Mandate-for-Many-Preventive-Services.pdf
4. Murphy C, Morris R, Johnson K, Rosenbaum S. Braidwood Management v Becerra could eliminate three quarters of the Affordable Care Act's most important preventive benefits for women, infants, and children. The Geiger Gibson Program in Community Health, The George Washington University; June 2023.
5. American Lung Association. Preventive services impacted by Braidwood v. Becerra. Published June 23, 2023. Accessed March 26, 2025. https://www.lung.org/getmedia/7ee72d9c-ee78-4a77-a419-9bfa87e69acd/Braidwood-Preventive-Services-Chart.pdf
6. United States Preventive Services Taskforce. A and B recommendations. Accessed March 26, 2025. https://www.uspreventiveservicestaskforce.org/uspstf/recommendation-topics/uspstf-a-and-b-recommendations
7. Blue Cross Blue Shield of North Dakota. Preventive health benefits and coding. Published 2024. Accessed March 26, 2025. https://www.bcbsnd.com/content/dam/bcbsnd/documents/general/Preventive-Coding-Guidelines.pdf
8. Blue Cross Blue Shield of North Carolina. Health care reform preventive services coding guide. Published 2024. Accessed March 26, 2025. https://www.bluecrossnc.com/content/dam/bcbsnc/pdf/providers/network-participation/bcbsnc-hcr-preventive-services-coding-guide.pdf
9. Cigna. A guide to Cigna's preventive health coverage: For health care professionals. Published 2016. Accessed March 26, 2025. https://www.mercyoptions.net/wp-

© 2025 Bronsard M et al. *JAMA Health Forum*.

content/uploads/2014/01/2016-Preventive-Health-Benefits-Guide-for-Health-Care-Professionals.pdf
10. Alliance Health. Procedure code lookup tool. Accessed March 26, 2025. https://www.alliancehealthplan.org/providers/procedure-code-lookup-tool/
11. STD Related Reproductive Health Training and Technical Assistance Center (STD TAC). List of ACA preventative services and CPT codes. Published 2016. Accessed March 26, 2025. https://www.ncsddc.org/wp-content/uploads/2019/09/List-of-ACA-Preventative-Services-and-CPT-Codes-_STDTAC1.pdf

© 2025 Bronsard M et al. *JAMA Health Forum*.
