# Supplementary material for: Use of No-Cost Preventive Services Jeopardized by Kennedy v Braidwood
Source: JAMA Health Forum. 2025 Apr 17;6(4):e251559. doi: 10.1001/jamahealthforum.2025.1559 (PMC12006864; doi:10.1001/jamahealthforum.2025.1559)
Supplement: Supplement 2. — Data Sharing Statement [file jamahealthforum-e251559-s002.pdf]

Data Sharing Statement

Bronsard. Use of No-Cost Preventive Services Jeopardized by Kennedy v Braidwood. *JAMA Health Forum*. Published April 17, 2025. doi:10.1001/jamahealthforum.2025.1559

Data
Data available: No

Additional Information
Explanation for why data not available: The analytic datasets used in this manuscript are not publicly available but were accessed by the authors under a restricted data use agreement between Stanford University and Merative. Code for both the cohort assembly and analysis are centrally stored and archived on the Stanford University Center for Population Health Sciences data portal. Investigators wishing to replicate this work should complete requirements to obtain MarketScan data access and request view-access to the project, including code and workflow, from Michelle Bronsard (bronsm@stanford.edu). Authors' code for cohort assembly and analysis is available on a public GitHub repository (github.com/PPML/preventive_services).
